# Serum calcium levels correlates with coronary artery disease outcomes

**DOI:** 10.1515/med-2020-0154

**Published:** 2020-11-09

**Authors:** Mian Wang, Shaodi Yan, Yong Peng, Yu Shi, Jiay-Yu Tsauo, Mao Chen

**Affiliations:** Department of Cardiology, West China Hospital, Sichuan University, No. 37, Guoxue Street, Chengdu, 610041, China; Department of Cardiology, Fuwai Hospital Chinese Academy of Medical Sciences, Shenzhen, Shenzhen, 518057, China; Shenzhen University School of Medicine, Shenzhen, 518060, China; Fuwai Hospital Chinese Academy of Medical Sciences, Shenzhen, Shenzhen, 518057, Guangdong Province, China; Department of Clinical Research and Epidemiology, Shenzhen Institute for Cardiovascular Disease, Shenzhen, 518057, Guangdong Province, China

**Keywords:** coronary artery disease, serum calcium, independent predictor, prognosis

## Abstract

**Background:**

Effect of serum calcium levels on prognosis of patients with coronary artery disease (CAD) is not well evaluated. We aimed to assess the associations of baseline serum calcium levels with both short-term and long-term outcomes in CAD patients.

**Methods:**

This study included 3,109 consecutive patients with angiographically confirmed CAD. Patients were categorized into quartiles according to admission serum calcium. Multivariable regression analysis was used to determine the association of serum calcium with mortality.

**Results:**

Compared to patients in the lowest quartile of serum calcium, patients in upper quartiles were presented with lower all-cause mortality (Hazard ratios [HRs] were −0.636 [95% CI: −0.424 to −0.954], −0.545 [95% CI: −0.351 to −0.846] and −0.641 [95% CI: −0.450 to −0.913] for three upper quartiles versus lowest quartile respectively), cardiovascular mortality (HRs 0.594 [0.368−0.961], 0.261 [0.124–0.551] and 0.407 [0.229–0.725]), and in-hospital mortality (Odd ratios [ORs] 0.391 [0.188–0.812], 0.231 [0.072–0.501] and 0.223 [0.093–0.534]). Consistent associations between serum calcium and long-term mortality were also obtained in subgroup analysis of ACS patients, stable CAD patients and discharged patients.

**Conclusions:**

Serum calcium is inversely associated with CAD and can independently predict both in-hospital and long-term mortality among CAD patients.

## 1 Introduction

Calcium serves an important role in cardiovascular disease [1,2]. Serum calcium directly contacts with and acts on blood cells and endothelial cells, and is also essential for maintaining extracellular calcium levels [3].

Serum calcium, a common clinical biochemical index, is a main component of extra-skeletal calcium. The prognostic value of serum calcium in coronary artery disease (CAD) has been evaluated in limited longitudinal studies that enrolled selective patients. Hypercalcemia was found to be an independent predictor of poor long-term outcomes in stable CAD patients surviving from a recent acute myocardial infarction [4]. Another study showed that hypocalcemia was closely associated with higher in-hospital mortality in patients of acute ST-elevation myocardial infarction [5]. These previous findings suggest that serum calcium is a potential prognostic factor of CAD and further study is warranted to more comprehensively assess the predictive value of serum calcium levels in CAD patients.

We therefore conducted this study to examine associations of baseline serum calcium levels with both in-hospital mortality and long-term outcomes in consecutive CAD patients with various clinical conditions ranging from stable coronary artery disease (SCAD) to acute coronary syndromes (ACS).

## 2 Patients and methods

### 2.1 Subjects

This study enrolled consecutive patients from July 2008 to September 2012. They were >18 years old. They all had CAD confirmed by angiography. The diagnosis of ACS was according to previously described criteria [6]. Stable CAD was diagnosed in the presence of chronic stable chest pain in patients having at least one >50% stenosis in the left main artery or >70% stenosis in proximal epicardial coronary artery (at least 1). Exclusion criteria: patients with pregnancy, severe gastrointestinal disease, malignancies, sever renal dysfunction (GFR < 30 mL/min), active bleeding, severe liver or haematological disorders, haemodynamic instability, or deficiency of serum calcium were excluded. This study was approved by the ethics committee. Written informed consents were obtained from all subjects.

### 2.2 Data collection

All subjects underwent a comprehensive medical history review, physical examination, and clinical chemistry analysis before enrollment. The collected data include: general information such as age, previous medical history, pre-hospital medications, in-hospital laboratory examination data, angiography, whether to receive PCI treatment, and in-hospital medication. Clinical chemistry included admission serum calcium, which was measured according to routine procedure, and, the first serum calcium level obtained during hospitalization. Hypertension was defined as a blood pressure higher than 140/90 mm Hg for two independent readings at least. Diabetes was diagnosed when fasting blood glucose level was >7.0 mmol/L twice.

### 2.3 Outcomes and follow up

The primary outcome of present study was all-cause mortality. Patients were followed for cardiac death and all-cause mortality. In-hospital death was also recorded. All outcomes were evaluated blindly by two independent investigators. The median follow-up time was 26 months (interquartile range 16–38). Follow-up was performed through contacting patients, their physicians, or family.

### 2.4 Statistical analyses

SPSS software (version 22.0) was used. Continuous variables, expressing as the mean ± standard deviation (SD), were analyzed with ANOVA. Categorical variables, presenting as counts and percentages, were compared with chi-square test. Cumulative survival plots for endpoints with different quartiles of serum calcium were evaluated by the Kaplan–Meier analysis and log-rank test. Univariate regression analysis was performed for all variables before multivariable analysis. The variables with *P* value less than 0.1 were enrolled in the following multivariable Cox or Logistic regression model. Multivariable Cox regression analysis was used to identify independent predictors of clinical outcomes at follow-up. In-hospital mortality was evaluated by multivariable logistic regression analysis. For continuous variables, we assessed the correlation between Schoenfeld residuals with time. For categorical variables, we depicted the plot of the log cumulative hazard versus time stratified by the variable of interest. The results showed that the age, PCI treatment and several in-hospital medications (including clopidogrel, statin and beta-blocker) violated the PH assumption in the model of the association with all-cause death in the overall patients, and PCI treatment violated the PH assumption in the model of the association with cardiovascular death in the overall patients, while beta-blocker treatment violated the PH assumption in the model of the association with cardiovascular death in the discharged patients. Then, in the multivariate model, we extended the model with adding the interaction variables, defined as each abovementioned variables multiply by time. Statistical significance was determined when *P* < 0.05.

## 3 Results

### 3.1 Patient characteristics

**Figure 1:**
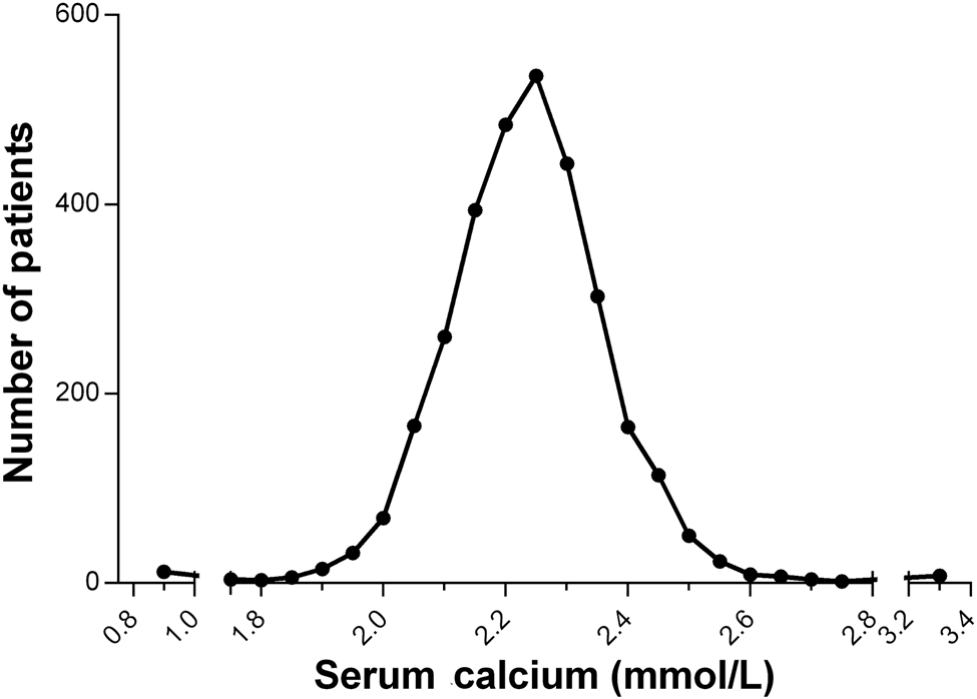
The distribution of mean admission serum calcium in overall participants. The mean admission calcium levels of 3,109 consecutive patients with angiographically confirmed CAD were collected. The distribution of the mean admission calcium levels was shown.

All 3109 CAD patients had complete follow-up data. Their clinical data is shown in Table 1. Their mean age was 64.41 ± 10.75 years and 79.41% of them were males. And, 71.7% presented with ACS, and 71.0% received PCI (percutaneous coronary intervention). The mean admission calcium level, which was normally distributed, was 2.20 ± 0.15 mmol/L (Figure 1). The admission calcium level in patients with ACS was lower than that in patients with stable CAD (2.201 vs 2.213 mmol/L, *p* = 0.036). The 81.4% of the calcium values were within the reference range (2.1–2.7 mmol/L). Based on the serum calcium upon admission, patients were divided into four quartiles (quartile 1: <2.12 mmol/L, quartile 2: 2.12 mmol/L to <2.21 mmol/L, quartile 3: 2.21 mmol/L to <2.28 mmol/L and quartile 4: >2.28 mmol/L).

**Table 1:** Baseline characteristics of overall participants and groups stratified by admission serum calcium levels

Variables	Overall	Quartile 1	Quartile 2	Quartile 3	Quartile 4	*P*-value
Serum calcium (mmol/L)		<2.12	2.12 to <2.21	2.21 to <2.28	≥2.28	
Number of patients	3,109	702	848	712	847	
Age (years)	64.41 ± 10.75	66.11 ± 10.28	64.92 ± 10.52	63.85 ± 10.80	62.97 ± 11.61	<0.001
Male, *n* (%)	2,469 (79.4)	576 (82.1)	696 (82.1)	549 (77.1)	648 (76.5)	0.004
Previous or current smokers, *n* (%)	1,855 (59.7)	434 (61.8)	520 (61.3)	420 (59.0)	481(56.8)	0.146
Hypertension, *n* (%)	1,717 (55.2)	375 (53.4)	439 (51.8)	406 (57.0)	497 (58.7)	0.018
Diabetes, *n* (%)	754 (24.3)	175 (24.9)	187 (22.1)	167 (23.5)	225 (26.6)	0.163
**Previous PCI or CABG,** ***n*** **(%)**						
PCI	362 (11.6)	77 (11.0)	104 (12.3)	96 (13.5)	85 (10.0)	0.165
CABG	35 (1.1)	6 (0.9)	9 (1.1)	7 (1.0)	13 (1.5)	0.594
**Previous drug treatment,** ***n*** **(%)**						
Aspirin	1,156 (37.2)	211 (30.1)	321 (37.9)	290 (40.7)	334 (39.4)	<0.001
Statin	770 (24.8)	140 (19.9)	201 (23.7)	214 (30.1)	215 (25.4)	<0.001
ACEI or ARBs	652 (21.0)	124 (17.7)	182 (21.5)	149 (20.9)	197 (23.3)	0.059
Beta-blocker	817 (26.3)	136 (19.4)	225 (26.5)	207 (29.1)	249 (29.4)	<0.001
BMI^a^	24.16 ± 2.92	24.01 ± 2.82	23.95 ± 2.83	24.27 ± 2.84	24.39 ± 3.12	0.006
**Blood pressure at admission (mmHg)**						
Systolic	130.35 ± 21.29	126.67 ± 23.33	129.38 ± 19.55	132.71 ± 21.17	132.40 ± 20.86	<0.001
Diastolic	76.58 ± 12.58	74.42 ± 13.34	75.76 ± 11.97	77.41 ± 12.72	78.49 ± 12.09	<0.001
Heart rate at admission (beats/min)	74.06 ± 13.86	75.58 ± 15.93	73.52 ± 13.38	73.17 ± 12.35	74.09 ± 13.63	0.006
Creatinine clearance^b^	70.28 ± 29.29	68.85 ± 24.82	69.94 ± 25.12	71.44 ± 40.68	70.82 ± 24.81	0.367
Total cholesterol (mmol/L)	4.09 ± 1.10	3.79 ± 0.96	4.02 ± 1.11	4.22 ± 1.11	4.28 ± 1.14	<0.001
LDL-cholesterol (mmol/L)	2.39 ± 0.94	2.24 ± 0.80	2.33 ± 0.95	2.45 ± 0.90	2.53 ± 1.03	<0.001
Triglyceride (mmol/L)	1.75 ± 1.13	1.47 ± 0.80	1.70 ± 1.19	1.79 ± 1.18	1.98 ± 1.20	<0.001
HDL-cholesterol (mmol/L)	1.15 ± 0.35	1.12 ± 0.42	1.13 ± 0.35	1.18 ± 0.34	1.17 ± 0.30	0.002
Serum potassium (mmol/L)	3.96 ± 0.45	3.85 ± 0.52	3.95 ± 0.42	3.99 ± 0.42	4.02 ± 0.44	<0.001
Serum sodium (mmol/L)	141.00 ± 3.63	140.20 ± 4.47	141.08 ± 3.22	141.38 ± 3.46	141.27 ± 3.28	<0.001
Serum chloride (mmol/L)	102.7 ± 18.15	105.47 ± 5.72	105.35 ± 3.28	104.67 ± 5.28	104.12 ± 5.78	<0.001
**Lesion characteristic,** ***n*** **(%)**						
Left main disease, *n* (%)	290 (9.3)	72 (10.3)	78 (9.2)	62 (8.7)	78 (9.2)	0.784
Triple vessel disease, *n* (%)	661 (21.3)	145 (20.7)	170 (20.0)	166 (23.3)	180 (21.3)	0.441
ACS, *n* (%)	2,229 (71.7)	534 (76.1)	601 (70.9)	491 (69.0)	603 (71.2)	0.22
PCI, *n* (%)	2,207 (71.0)	511 (72.8)	599 (70.6)	490 (68.8)	607 (71.7)	0.396
**Drug treatment in hospital and during follow-up,** ***n*** **(%)**						
Aspirin	2,954 (95.0)	665 (94.7)	808 (95.3)	677 (95.1)	804 (94.9)	0.965
Clopidogrel	2,866 (92.2)	624 (92.2)	779 (91.9)	657 (92.3)	783 (92.4)	0.976
Statin	2,890 (92.2)	655 (93.4)	786 (92.7)	667 (93.7)	782 (92.3)	0.699
ACEI or ARBs	1,843 (59.3)	385 (54.8)	491 (58.0)	435 (61.1)	532 (62.8)	0.009
Beta-blocker	2,123 (68.3)	429 (61.1)	559 (66.0)	506 (71.1)	629 (74.3)	<0.001
Nitrated derivative	1,399 (45.0)	281 (40.0)	394 (46.5)	329 (46.2)	395 (46.6)	0.028

Data are expressed as mean ± SD or counts and percentages, as appropriate. ACEI: angiotensin-converting enzyme inhibitors, ACS: acute coronary syndrome, ARBs: angiotensin-receptor blockers, CABG: coronary-artery bypass grafting, PCI: percutaneous coronary intervention.

^a^Body mass index is the weight in kilograms divided by the square of the height in meters.

^b^Creatinine clearance was calculated using the Cockcroft–Gault equation (mL/min).

The previous history of patients including hypertension, diabetes mellitus, smoking history, prior PCI, and prior coronary-artery bypass grafting (CABG) did not differ among the quartiles. And there was no significant difference in admission creatinine clearance rate, lesion characteristics, clinical presentation (ACS or stable CAD), anti-platelet treatment (aspirin and clopidogrel), statin treatment, and PCI treatment. Patients in the lowest quartile of serum calcium were more likely to be presented with higher age, higher heart rate and lower blood pressure, as well as a lower level of albumin, total cholesterol, low density lipoprotein cholesterol, serum sodium and serum potassium.

### 3.2 Outcomes in consecutive patients

**Figure 2:**
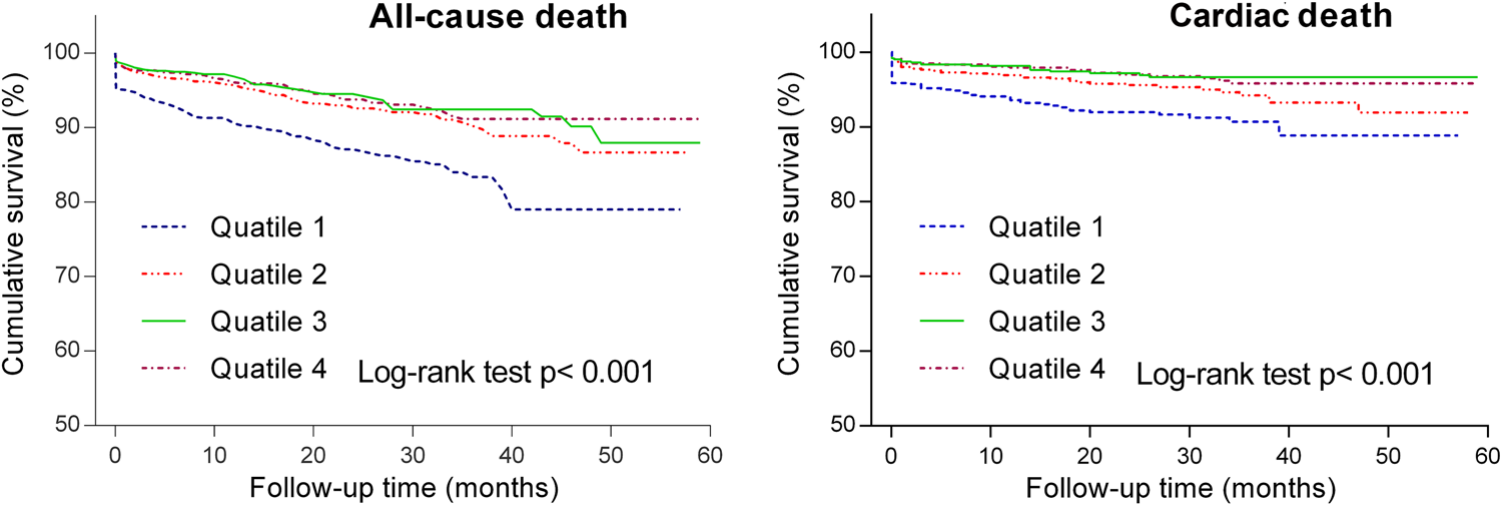
Kaplan–Meier curves for the all-cause and cardiac mortality in overall participants according to admission serum calcium levels. Based on the serum calcium upon admission, patients were divided into four quartiles of quartile 1: <2.12 mmol/L, quartile 2: 2.12 mmol/L to <2.21 mmol/L, quartile 3: 2.21 mmol/L to <2.28 mmol/L and quartile 4: >2.28 mmol/L.

**Figure 3:**
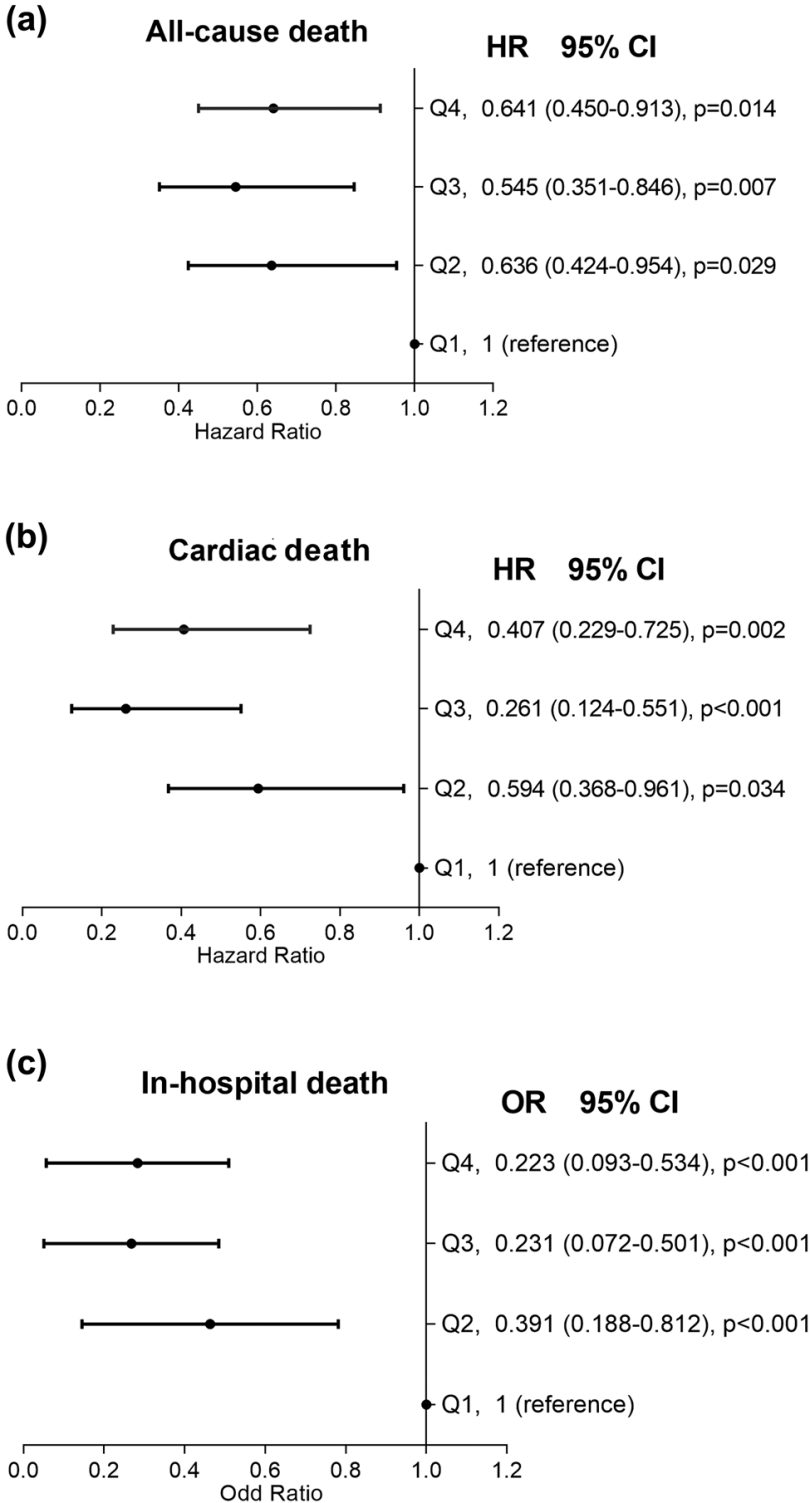
Results of multivariate logistic regression analysis for the all-cause (a) and cardiac mortality (b) and results of multivariate Cox regression analysis for the in-hospital mortality (c) in overall participants according to admission serum calcium levels. Hazard ratios were adjusted for all corresponding factors whose p value less than 0.1 listed in Table 2. Based on the serum calcium upon admission, patients were divided into four quartiles of quartile 1: <2.12 mmol/L, quartile 2: 2.12 mmol/L to <2.21 mmol/L, quartile 3: 2.21 mmol/L to <2.28 mmol/L and quartile 4: >2.28 mmol/L.

A total of 259 deaths, including 58 in-hospital deaths, occurred during a median follow-up period of 26 months (interquartile range 16–38). Kaplan–Meier analysis and log-rank test were used to analyze the cumulative survival of patients with different quartiles of serum calcium. The observed long-term rates of all-cause death and cardiovascular death were significantly higher in the lowest calcium quartile than others as shown in Figure 2. Univariate regression analysis results of each baseline characteristics were listed in Table 2 and all variables whose *P* value less than 0.1 were enrolled in the following multivariable Cox regression model. After adjustment for baseline differences with multivariable analysis, the risks of all-cause death (Figure 3a), cardiac death (Figure 3b) and in-hospital death (Figure 3c) remained consistently higher in the lowest quartile. Multivariable logistic analysis showed that after adjusting for potential confounding variables, low level of serum calcium was also an independent predictor of in-hospital mortality.

**Table 2:** Univariate regression analyses of factors associated all-cause mortality, cardiac cause mortality and in-hospital mortality

Factors	All-cause death		CV death		In-hospital death	
	Unadjusted HR	*P*-value	Unadjusted HR	*P*-value	Unadjusted OR	*P*-value
Age	1.068^#^	<0.001	1.051	<0.001	1.041	0.004
Male	0.826	0.193	0.794	0.235	1.757	0.050
Hypertension	1.120	0.369	1.079	0.653	0.019	<0.001
Diabetes	1.725	<0.001	1.856	<0.001	2.244	0.003
Previous PCI	0.464	0.004	0.271	0.004	0.267	0.067
Previous aspirin	0.646	0.002	0.513	<0.001	0.482	0.021
Previous statin	0.521	<0.001	0.434	<0.001	0.412	0.028
Previous beta-blocker	0.556	<0.001	0.410	<0.001	0.007	<0.001
BMI^a^	0.923	<0.001	0.916	0.003	0.957	0.343
SBP	0.996	0.178	0.992	0.061	0.981	0.003
DBP	0.989	0.036	0.989	0.110	0.975	0.020
Creatinine clearance^b^	0.970	<0.001	0.972	<0.001	0.960	<0.001
Albumin	0.906	<0.001	0.921	<0.001	0.920	0.003
LDL-cholesterol	1.106	0.112	1.236	0.006	1.211	0.125
Triglyceride	1.007	0.094	0.812	0.040	0.864	0.344
HDL-cholesterol	1.387	0.010	1.054	0.827	0.726	0.488
Serum potassium	1.080	0.010	0.908	0.606	1.476	0.152
Serum sodium	0.924	<0.001	0.939	<0.001	0.943	0.028
Serum chloride	0.980	0.001	0.981	0.020	0.972	0.008
Left main disease	1.868	<0.001	2.281	<0.001	2.325	0.013
Triple vessel disease	2.275	<0.001	2.396	<0.001	2.675	<0.001
ACS	1.568	0.004	1.903	0.004	11.313	0.001
PCI	0.700^#^	0.006	0.661^#^	0.016	1.579	0.162
Aspirin	0.409	<0.001	0.572	0.075	0.020	<0.001
Clopidogrel	0.707	0.083	0.860	0.605	0.021	<0.001
Statin	0.709^#^	0.096	1.329	0.434	0.020	<0.001
ACEI or ARBs	0.779	0.045	0.774	0.126	0.014	<0.001
Beta-blocker	0.722^#^	0.011	0.774	0.139	0.021	<0.001
Nitrated derivative	0.990	0.939	1.032	0.849	0.021	<0.001

^#^Relative risk at *t* = 0.

ACEI: angiotensin-converting enzyme inhibitors, ACS: acute coronary syndrome, ARBs: angiotensin-receptor blockers, DBP: diastolic blood pressure, HR: hazard ratio, OR: odd ratio, PCI: percutaneous coronary intervention, SBP: systolic blood pressure.

^a^Body mass index is the weight in kilograms divided by the square of the height in meters.

^b^Creatinine clearance was calculated using the Cockcroft–Gault equation (mL/min).

### 3.3 Outcomes in subgroups

**Figure 4:**
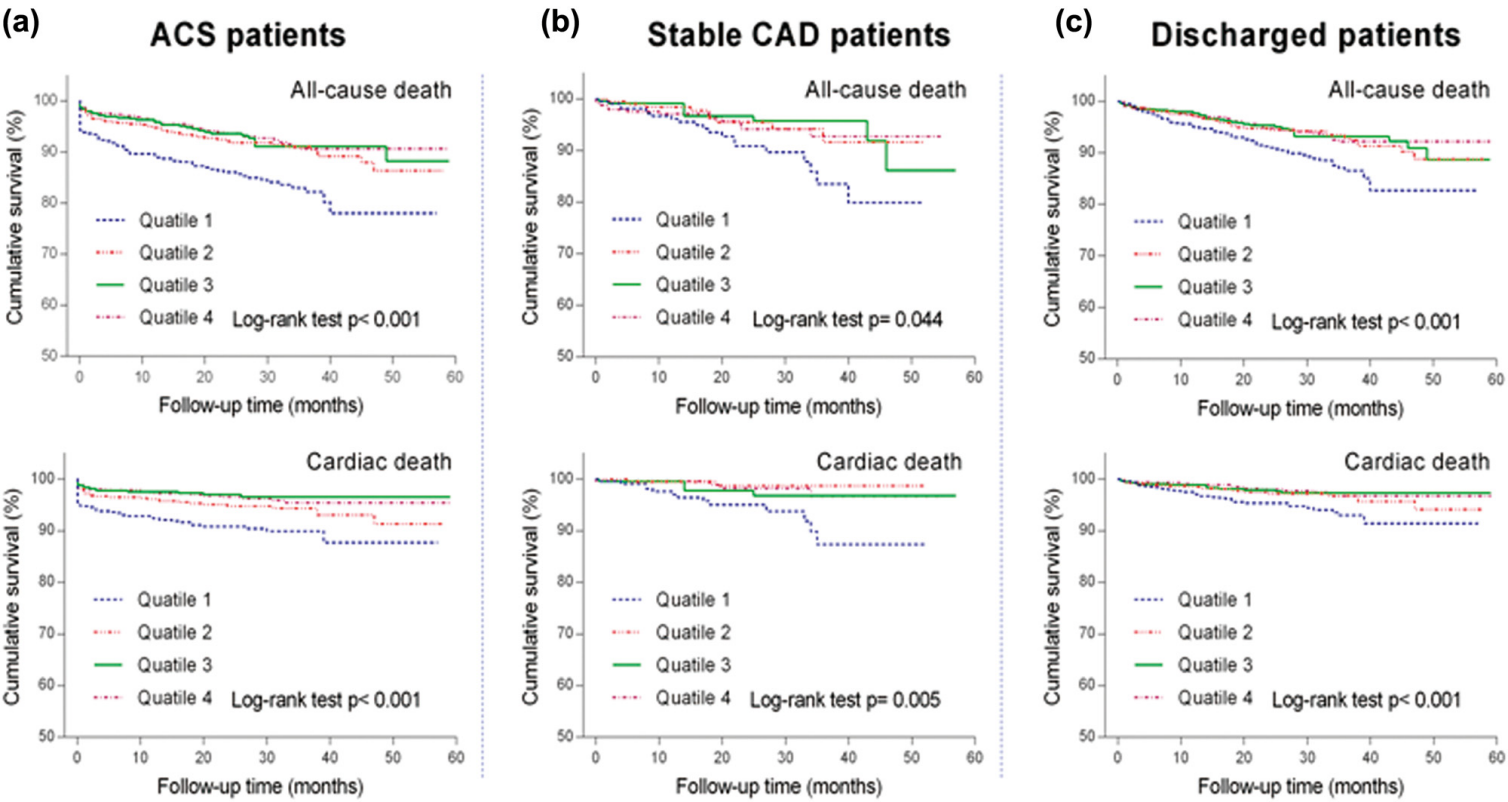
Kaplan–Meier curves for the all-cause and cardiac mortality in ACS patients (a), stable CAD patients (b) and discharged patients (c), respectively. Based on the serum calcium upon admission, patients were divided into four quartiles of quartile 1: <2.12 mmol/L, quartile 2: 2.12 mmol/L to <2.21 mmol/L, quartile 3: 2.21 mmol/L to <2.28 mmol/L and quartile 4: >2.28 mmol/L.

**Figure 5:**
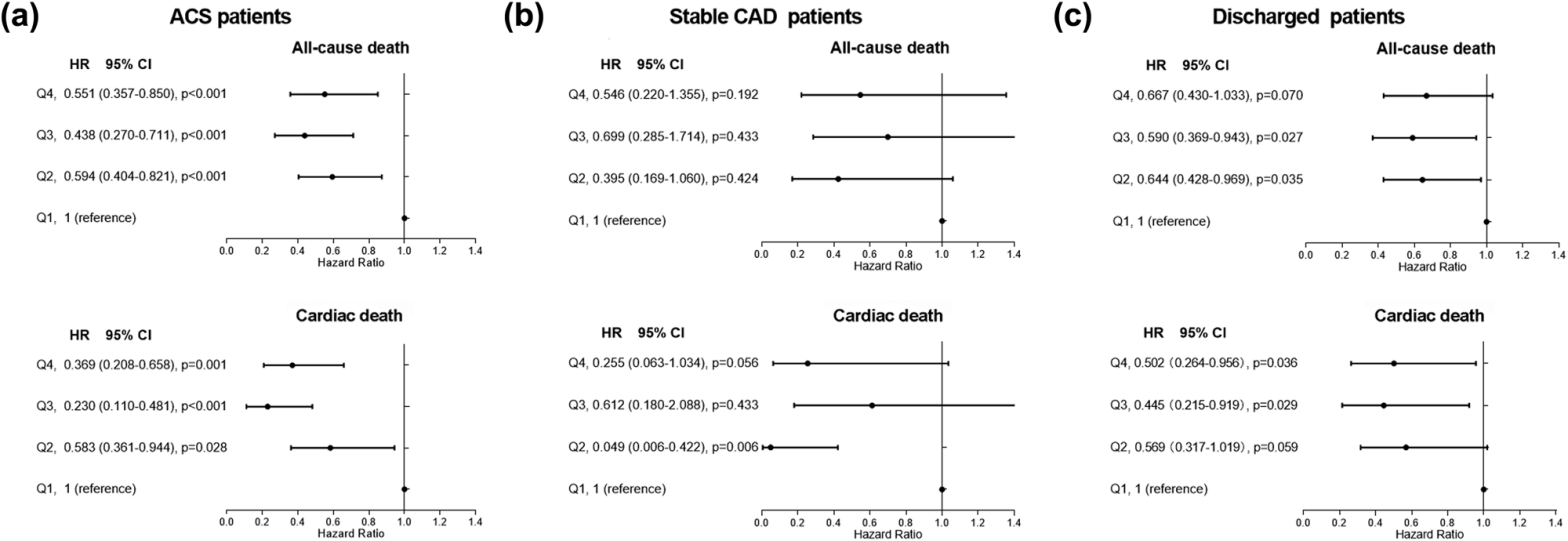
Results of multivariate Cox regression analysis for the all-cause and cardiac mortality in each subgroup. (a) ACS patients; (b) stable CAD patients; (c) discharged patients. Hazard ratios of each subgroup was adjusted for all corresponding factors whose *p* value less than 0.1 listed in Table 3. Based on the serum calcium upon admission, patients were divided into four quartiles of quartile 1: <2.12 mmol/L, quartile 2: 2.12 mmol/L to <2.21 mmol/L, quartile 3: 2.21 mmol/L to <2.28 mmol/L and quartile 4: >2.28 mmol/L.

We also performed survival analysis in the subgroups of ACS patients, stable CAD patients and discharged patients. Consistent with the results of unadjusted analysis for overall patients, the lowest calcium quartile showed significantly higher all-cause mortality and cardiac cause mortality in each subgroup as illustrated in Kaplan–Meier survival curves (Figure 4). Table 3 shows univariate Cox regression analyses results of factors associated with both all-cause and cardiac mortality. Higher mortality in discharged patients and ACS patients with lowest quartile of calcium persisted after adjustment for multiple confounders (Figure 5).

**Table 3:** Univariate regression analyses of factors associated long-term outcome (all-cause mortality and cardiac mortality) in ACS patients, stable CAD patients and discharged patients

Facters	ACS patients				Stable CAD patients				Discharged patients			
	All-cause death		CV death		All-cause death		CV death		All-cause death		CV death	
	HR	*P*-value	HR	*P*-value	HR	*P*-value	HR	*P*-value	HR	*P*-value	HR	*P*-value
Age	1.070	<0.001	1.052	<0.001	1.059	<0.001	1.047	0.036	1.078	0.001	1.062	<0.001
Male	0.756	0.079	0.685	0.064	1.361	0.425	0.363	0.170	0.930	0.675	1.052	0.846
Diabetes	1.797	<0.001	1.988	<0.001	1.378	0.309	1.183	0.721	1.602	0.002	1.535	0.053
Previous PCI	0.491	0.049	0.211	0.029	0.271	0.004	0.486	0.243	0.521	0.023	0.338	0.033
Previous aspirin	0.658	0.009	0.530	0.005	0.747	0.309	0.633	0.278	0.695	0.018	0.540	0.010
Previous statin	0.449	<0.001	0.381	0.002	0.892	0.708	0.810	0.638	0.781	0.165	0.494	0.018
Beta-blocker	0.622	0.010	0.504	0.001	0.422	0.019	0.183	0.021	0.625	0.009	0.467	0.009
BMIa	0.935	0.006	0.944	0.069	0.860	0.003	0.772	0.001	0.913	0.343	0.892	0.002
Grace score	1.026	<0.001	1.021	<0.001	—	—	—	—	—	—	—	—
DBP	0.984	0.005	0.987	0.073	1.015	0.212	1.007	0.697	0.993	0.254	0.998	0.782
Heart rate	1.028	<0.001	1.030	<0.001	1.033	<0.001	1.034	0.004	1.023	<0.001	1.027	<0.001
Creatinine clearanceb	0.971	<0.001	0.974	<0.001	0.970	<0.001	0.960	<0.001	0.973	<0.001	0.976	<0.001
Albumin	0.910	<0.001	0.927	<0.001	0.900	0.001	0.909	0.035	0.901	<0.001	0.923	<0.001
LDL-cholesterol	1.029	0.698	1.176	0.067	1.355	0.020	1.424	0.047	1.075	0.326	1.203	0.064
Triglyceride	0.794	0.006	0.840	0.092	1.008	0.026	0.501	0.069	1.008	0.061	0.774	0.050
HDL-cholesterol	1.303	0.060	0.942	0.828	2.489	0.038	2.283	0.199	1.506	0.001	1.226	0.426
Serum sodium	0.945	<0.001	0.939	<0.001	0.941	0.069	0.965	0.539	0.941	<0.001	0.938	0.001
Serum chloride	0.980	0.001	0.981	0.023	0.988	0.542	0.993	0.814	0.983	0.020	0.993	0.667
Left main disease	2.020	<0.001	2.510	<0.001	0.913	0.879	0.622	0.642	1.749	0.006	2.402	0.001
Triple vessel disease	2.132	<0.001	2.147	<0.001	2.799	0.002	3.638	0.004	2.180	<0.001	2.290	<0.001
PCI	0.612	0.001	0.558	0.003	0.644	0.128	0.582	0.199	0.575	<0.001	0.455	<0.001
Aspirin	0.426	0.002	0.743	0.515	0.279	<0.001	0.265	0.005	0.294	<0.001	0.331	<0.001
Clopidogrel	0.795	0.478	0.942	0.897	0.437	0.005	0.490	0.100	0.531	0.002	0.528	0.033
Statin	0.894	0.676	1.567	0.325	0.383	0.005	0.786	0.697	0.540	0.003	0.847	0.654
ACEI or ARBs	0.792	0.092	0.771	0.155	0.741	0.290	0.819	0.626	0.859	0.284	0.884	0.555
Beta-blocker	0.749	0.043	0.808	0.262	0.625	0.100	0.637	0.276	0.616	<0.001	0.615^#^	0.021

^#^Relative risk at *t* = 0.

ACEI: angiotensin-converting enzyme inhibitors, ARBs: angiotensin-receptor blockers, DBP: diastolic blood pressure, HR: hazard ratio, PCI: percutaneous coronary intervention, SBP: systolic blood pressure.

^a^Body mass index is the weight in kilograms divided by the square of the height in meters.

^b^Creatinine clearance was calculated using the Cockcroft-Gault equation (mL/min).

## 4 Discussion

This present study evaluated prognostic values of baseline serum calcium in consecutive CAD patients and suggests a significantly higher all-cause and cardiac mortality in subjects with lower serum calcium. Higher in-hospital mortality was found in patients with lower admission serum calcium, most of whom were ACS patients (56/58), further confirming the result of a recent study by Lu et al. conducted in patients with acute myocardial infarction [5].

Furthermore, not only predicting short-term death (in-hospital mortality), lower level of serum calcium is also an independent predictor for mortality in long-term follow-up period, which was confirmed in overall participants, ACS patients and also discharged patients. In stable CAD patients, slightly significant higher all-cause mortality in the lowest quartile versus the upper quartiles in unadjusted analysis was attenuated after full confounder adjustment, which may be partly due to relatively small amount of subjects. These results of calcium level in predicting long term mortality seems contradictory to previous studies [4,7,8] at first sight, for previously hypercalcaemia but not hypocalcaemia was believed to be a predictor of higher mortality. But after a second thought, this present study is actually a beneficial supplement for previous findings. Firstly, all subjects enrolled in our study were hospitalized patients with angiography confirmed CAD but not community population as in previous studies [7,8]. Though the population in Grandi’s study [4] was CAD patients, the baseline data of patients was collected weeks after discharge and in hospital death was naturally excluded. Secondly, serum calcium level in our study was obviously lower than previous studies. Serum calcium greater than 2.45 mmol/L was associated with a significantly increased mortality compared with others in Leifsson’s study [7], but only 2.8% subjects had this calcium level in this present study. The lower cut off value of serum calcium in Grandi ‘s study [4] was even higher than the upper cut off value in our study. Considering different races and different life style, such as the daily intake of calcium in China is much lower than that in European and North American countries [9], this discrepancy may be also due to different study population. A combination of our study with previous studies strongly suggests serum calcium may show a U-shaped relationship with mortality just as potassium [10,11]. This is supported by a recent clinical study enrolling patients with ACS by Gu et al. [12]. The patients’ serum calcium level in Gu’s study [12] was slightly higher than those in our study, and their results suggested a reverse J-shaped association between serum calcium levels and 21.8-month mortality. Thirdly, Grandi’s study [4] observed considerably higher serum calcium level of patients in rehabilitation after a recent acute coronary event than other studies, and our study showed lower admission calcium level in patients with ACS than others with stable CAD. These differences suggest potential dynamic change of serum calcium in different CAD periods. Finally, previous study has shown that lower level of serum calcium is an independent predictor of all-cause mortality [12]. This present study further refined the association of hypocalcemia and higher cardiovascular mortality, and expanded the subjects from ACS patients to CAD patients. Further researches are required to clarify the underlying mechanism of the relationship between hypocalcemia and adverse outcomes of such patients.

The underlying mechanisms that account for the association between the baseline serum calcium levels and mortality with CAD remain unclear. Potentially, decreased serum calcium may disturb cardiac electrophysiological activity. For cardiomyocytes, low level of serum calcium may delay the closure of calcium channel, thus extending the plateau phase of the cardiac action potential [13], which is widely accepted as an independent high risk factor for mortality [14]. Additionally, calcium deficiency may impair vascular smooth muscle cells, increase blood pressure and disturb lipid metabolism, all of which could aggravate cardiovascular condition and worse the prognosis of CAD patients [5,15,16].

Calcium supplement, alone or combined with vitamin D, shows potential effects on cardiovascular events in a large number of studies, but the results of these studies are still in controversial and not yet sufficient to get a certain conclusion [17,18,19,20,21]. A recent prospective cohort study demonstrated that the use of calcium tablets was associated with all-cause mortality only in male individuals with a dietary calcium intake of >1,400 mg/day [22]. A meta-analysis found that in those with low dietary calcium intake (<700 mg/d), calcium supplement was associated with a reduction in stroke risk; whereas an increased risk of stroke was observed in those with high dietary calcium intake [23]. These results indicate that the effects of calcium supplement are probably population dependent. Thus, whether proper calcium supplement therapy especially for CAD patients with lower level of serum calcium would improve their prognosis is a matter worthy of further study.

For a more comprehensive assessment of serum calcium, we enrolled various clinical subtypes of CAD and adjusted most possible factors in multivariable regression model, but there are still some limitations should be taken into account in the interpretation of our analysis. First, the data of hormones that regulate serum calcium as well as diuretic therapy and dietary calcium intake were not collected, which have been widely reported as potential factors affecting cardiovascular diseases and may affect patients’ calcium level [24,25]. For an individual, the serum calcium concentration is stable, which facilitates and validates its measurement in this real-world observational study [26], but calcium related hormones fluctuate in a wide range to maintain calcium homeostasis, which limited their application in this present study. Second, due to the inherent limitation of real-world studies, we can hardly reach the multiple measurement of serum calcium in most patients. Thus, the dynamic change of serum calcium during follow-up was not collected. Third, given enrolled consecutive patients, populations in this single-center study were always with relatively lower serum calcium and thus the prognostic value of hypercalcaemia was not assessed. Fourth, the use of many confounding variables in multivariate analysis could have created bias. All these issues mentioned above should be addressed by future well-designed trials to confirm whether serum calcium is an independent actor, one of co participants or just a standby in the pathogenesis of CAD.

## 5 Conclusions

The results of this study suggest low serum calcium level is an independent predictor for mortality in CAD patients. Further studies are warranted to determine underlying causal pathways and whether patients with hypocalcaemia could benefit from calcium supplement.
